# Human Development XI: The Structure of the Cerebral Cortex. Are There Really Modules in the Brain?

**DOI:** 10.1100/tsw.2007.256

**Published:** 2007-12-10

**Authors:** Tyge Dahl Hermansen, Søren Ventegodt, Isack Kandel

**Affiliations:** ^1^ Quality of Life Research Center, Teglgårdstræde 4-8, DK-1452 Copenhagen K, Denmark; ^2^ Research Clinic for Holistic Medicine, Copenhagen, Denmark; ^3^ Nordic School of Holistic Medicine, Copenhagen, Denmark; ^4^ Scandinavian Foundation for Holistic Medicine, Sandvika, Norway; ^5^ Interuniversity College, Graz, Austria; ^6^ Faculty of Social Sciences, Department of Behavioral Sciences, Ariel University Center of Samaria, Ariel, Israel; ^7^ National Institute of Child Health and Human Development, Jerusalem, Israel

**Keywords:** holistic biology, theoretical biology, clinical holistic medicine, public health, neurobiology, cortex, brain

## Abstract

The structure of human consciousness is thought to be closely connected to the structure of cerebral cortex. One of the most appreciated concepts in this regard is the Szanthagothei model of a modular building of neo-cortex. The modules are believed to organize brain activity pretty much like a computer. We looked at examples in the literature and argue that there is no significant evidence that supports Szanthagothei's model. We discuss the use of the limited genetic information, the corticocortical afferents termination and the columns in primary sensory cortex as arguments for the existence of the cortex-module. Further, we discuss the results of experiments with Luminization Microscopy (LM) colouration of myalinized fibres, in which vertical bundles of afferent/efferent fibres that could support the cortex module are identified. We conclude that sensory maps seem not to be an expression for simple specific connectivity, but rather to be functional defined. We also conclude that evidence for the existence of the postulated module or column does not exist in the discussed material. This opens up for an important discussion of the brain as functionally directed by biological information (information-directed self-organisation), and for consciousness being closely linked to the structure of the universe at large. Consciousness is thus not a local phenomena limited to the brain, but a much more global phenomena connected to the wholeness of the world.

### INTRODUCTION

In our quest for a new understanding of the mysterious connection between life, matter and consciousness[1-10], we have now turned our interest towards the human brain. The structure held responsible for the structure and quality of human consciousness is the neo-cortex, the only structure that discriminates us qualitatively from the other vertebrates. Our motivation for the exploration of the life-matter-consciousness-link is our finding that quality of life, health and ability primarily is determined by our consciousness. This discovery has lead us to the interesting possibility that guided shifts in the state of consciousness and the development of self-insight with a more positive, responsible and constructive philosophy of life can be used as medicine to help cure patients. Based on this strategy we have made a series of papers setting the strategy for alleviating healing many different physical, mental, existential, and sexual diseases and health problems[11-52]. Another motivation for a deep exploration of the matter-life-consciousness link is the strange fact that “holistic healing” - the healing of the patients total life and existence, including body, mind and spirit – seems to be closely connected to the patient recovering his experience of “sense of coherence” (SOC)[53-59]. SOC is actually the experience that one conscious being is connected to the whole universe though our physical and mental existence[59]; to bring the patient back to be an integrated part of the world seems to be the fundamental idea of all medicine, the tratition going all the way back to Hippocrates and his students[60].

Many theories concerning the neo-cortical function are based on the possible existence of discrete modules in the neo-cortex as suggested by Szentagothai[61]. It is tempting to do this, because the function of neo-cortex is a lot easier to understand and modulate in a mathematical way. Unfortunately, evidence does not seem to exist concerning modules in Szentagothai’s understanding, as approximately 300 μ large, discrete columns that are able to make the “building stones” of association-cortex. In this paper, we discuss the possibility of the existence of Szentagothai’s cortical modules.

### Does Evidence for Szentagothai’s Cortex Module Exist?

Szentagothai[62], wrote "It was then (1974) that the assembly of larger tissue complexes from repetitive units of similar build - not unlike the integrated circuits in electronics technology - became an attractive conceptual model to explain how such an immense complexity in "wiring" might be put together without having to make unrealistic demands on the genetic apparatus responsible for this feat in system engineering". Szentagothai had both the genes and the microchips in his mind, when he introduced the modular concept into the association cortex. He realized that repetition of a structure coded by the genes as basis of the cortex development would economize the specific amount of information delivered by the genes. Even if we do not know of any molecular mechanism of such kind, this is a good idea, because such copy mechanism does not deal with DNA replication. Instead it deals with supra cellular patterns. Therefore, we have gradually gotten confident with the description of such phenomena through the conception of positional information. Analogue examples such as the development of testine-villi may be described in this way and therefore modules are an attractive idea. Furthermore, genes can be thought to deliver “continuous modules” (as in visual cortex, see later), and maybe create connectivity in a closely netted, unlimited, roomy way.

About an experiment[61,63] invented to prove that association cortex is constructed by discrete modules Szentagothai[61] wrote, “Here at last is unambiguous evidence for really columnar cortical architecture”. In this experiment, proteins containing injected 3H-amino acids were transported to cortical areas of the brain. After subsequent auto-photography, these experiments unveiled the finest 300 μ broad vertical columns in all layers of cortex[62,63,64]. Szentagothai[62] wrote, "It is most interesting to compare the overall size of the columns with the arborisation pattern of individual cortico-cortical afferents as it appears in the Golgi picture. Majorossy shows that both the size and the shape of the arborisation space correspond very closely to the columns, as delineated by the degeneration or auto-radiographic tracing techniques". Consequently, this column is analogues to the termination column of the corticocortical afferent. The big question then, is if the identified 300 μ broad termination column represents the module Szentagothai tries to prove the existence of. Seemingly, he has not found evidence enough to support such kind of structure[61,62].

Szentagothai supposed that this area had a great convergence. But Goldman and Rakic[63] said: “Such clear modular pictures as those of the figure[63] are not often seen, because the injections necessarily must cover many modules that result in agglomerations of the labelled modules, often in strips". This indicates an overlap between the termination columns and why fully discrete modules seem not to exist. But, of cause, a "half discrete" module represented by the largest concentration of afferents, cannot be excluded on the basis of this.

In cortex about 2,500,000,000 corticocortical afferents have been identified by Kandel et al[65]. But cortex has not room for more than approx. 3,000,000 columns of 300 μ in diameter, only enough to supply approx. 100 afferents to each column. If then a discrete module exists, a great amount of afferents may terminate within this. Therefore, it does not seem reasonable to take the single afferents termination, as indication for the existence of superior modules.

Szentagothai supports his opinion, that the primary sensory cortex has a columnar structure, on evidences from early research[62,65,67]. He emphasized two examples from respectively the somatic sensory cortex, 1), and the visual cortex, 2), that he thinks corresponds to the potential modules of the association cortex. However, he realized that the modules are not evident in association cortex.

1) *Barrels:* In a rodent's brain, "barrels" exists in the fourth layer of the somatic sensory cortex. Each barrel contains 2500 neurons arranged around a hollow centre. 100 somatic afferent fibres lead to these barrels from receptors around the barrels. If a barrel is removed, the other barrels are regularly distributed and fill out the area where the barrel is missing. This arrangement reminds us of the primates somatic sensory cortex, where inputs also are recorded in the fourth layer. If some nerve treads are damaged in this layer, a corresponding re-arrangement to that of the barrels can be realized in this layer, see[65].

Because a rodent only has 50-100 barrels each delivering huge amounts of information, it seems reasonable that each barrel has an individual discrete representation in cortex. This is a unique exception of the organization of the somatic sensory cortex[65]. Such kind of organization of the representation only exists in the fourth layer of the somatic sensory cortex, why it cannot be explained as a cortical column. Because the amount of barrels can vary independent of the area of the modality, it can be stated that if the amount of barrels determines the size of cortex, a single barrel cannot be a structural module.

2) *Hyper columns:* The hyper columns are found in primary visual cortex[65]. In one direction these are organized in ocular dominance columns, and in the other direction they are organized in orientation columns. However, we do not think the expression column fits here, because the ocular dominance column is defined by the termination of the afferent in the fourth layer where they do not make discrete columns, but instead alternate stripes from right and left eye. Perpendicular to these, the orientation columns can be defined as serial prisms that separately can be adjusted so their angle is a bit displaced compared with the prism. But nothing indicates that the line detector is not continuous. Also, the hyper column is arbitrary defined as a 1-2 mm large part of the cortex that makes up a right and a left stripe on the one side, and a complete round of the line detector on the other. The colour modality are handled in "blobs" in layer two and three of cortex[68]. Therefore, it is not likely that they represent the cortical module. Generally, for instance concerning the receptive field[65], all cells are identical in a very thin layer of the somatic sensory cortex. Possible, this field corresponds to the "micro-columns" described by [62,69], of approx. 10-30 μ, but not to the expected "micro column".

Maybe, cortex is constructed by continuous "modules" as the hyper column that very well could be a repeated structure coded by the genes, and seemingly, Szentagothai did not find any support for his discrete modules in this possibility, because the primary sensory cortex did not show any characteristics of a discrete modular structure. Also in the primary motor cortex the evidence for the presence of columns is weak (Hultborn, Personally communication), because the structure of stripes can only be distinguished here, where the organization seems much more to look like the visual cortex, analogue to the ocular dominance stripes, see [70].

### Other Data That Could Support The Existence Of A Cortex Module

In colouration experiments with myalinized fibres, vertical bundles of afferent- and/or efferent-fibres are often seen many places in the cortex as reviewed by Williams and Warwick[71]. In LM, the distance between these is approx. 2-3 pyramid cell diameter (20-150 μ) and approx. 10 μ in thickness, and seemingly 10-20 of these myalinized axons are seen to loose the myalinization throughout the cortex (own observations). These "weigot-columns" are hardly Szentagothai's modules, because 1) the distance between these is too small and 2) each of the large modules should receive 1000 afferents and deliver 1,000 efferents, and not 10-20. But maybe these10-20 myalinized axons correspond to "micro-columns" of 5 μ thickness described by Mountcastle[72].

It seems like the outspread termination of mono-aminergic fibres in neo-cortex can be observed as a gap of around 100 μ[73]. This distance is too little to fit with modules of 300 μ. But other researchers think that cortex contains non-modular "overlapping columns" of 800 μ[72].

### Sensory Maps Seems Not To Be An Expression For Simple Specific Connectivity

The somatotope map shows immediately reorganization[65], when the supplying nerve is cut. This shows that the afferents terminations happen through a large area, and do not indicate that they are "hard wired" in a simple way. We can guess that such phenomena are caused by complex connectivity, and find it obvious to set the organization of the map in connection with the attention and the positional information that manage the complex dynamics of the morphogenesis.

In the same way, because the thalamus-cortical afferents terminates opposite large areas, the ocular dominance columns seem to make up a kind of functional organizations[64]. Szentagothai[61] realized this and proposed that inhibitory inter-neurons adjust the afferents. But such arrangement does not support the existence of structural discrete columns in the sensory cortex.

## DISCUSSION

### Implications for Holistic Medicine

Millennia ago, around 300 BC, at the island of Cos in old Greece, the students of the famous physician Hippocrates[60] worked to help their patients to step into character, get direction in life, and use their human talents for the benefit of their surrounding world. For all that we know this approach was extremely efficient medicine that helped the patients to recover health, quality of life, and ability, which resulted in Hippocrates attaining great fame. For more than 2000 years this was what medicine was about in most of Europe.

On other continents similar medical systems were developed. The medicine wheel of the native Americans, the African Sangoma culture, the Samic Shamans of northern Europe, the healers of the Australian Aboriginals, the ayurvedic doctors of India, the acupuncturists of China, and the herbal doctors of Tibet all seems to be fundamentally character medicine, re-connecting man to his world.

Interestingly, the Hippocratic and the transcultural medical traditions gave birth to two succesfull movements in the last century: psychoanalysis[74,75] and psychodynamic therapy[76,77], developed further into today's clinical holistic medicine, integrating "short-term psychodynamic psychotherapy" (STPP)[78,79]) with the many traditions of bodywork (developed further into emotionally realizing bodywork by Reich[80], Lowen[81] and Rosen[82]) and existential work, today often much inspired by Antonovsky and the concept of SOC [83,84].

What we have learned by following this long journey of medicine that the grand medical heritage from the planet's different cultures teach us to work on body, mind and spirit at the same time; medicine men of all kinds have always combined talking, touching, and praying.

The fundamental problem of understanding the connection of human consciousness and health is this: how do we understand the experience of true connectedness with the universe of a human being, if the brain is just a computer generating the consciousness? The module concept reduces the brain from being a pattern-formatting organ under information directed control from the wholeness of the being and the wholeness of the world, into just being a computer with a pretty isolated function.

The importance of the module concept is seen in the attractiveness of this concept, which gives a feeling of understanding the brain, at least to a certain level, as a computer, or network of computers - the modules, in many models working very much like central processing units (CPUs). If there are not modules and not real structure of the cerebral cortex, only billions of brain-cells firing at their own will, a highly structured consciousness really is a true mystery. This mystery opens up for the possibility that our consciousness is structured true our complex interaction with the surrounding world on an informational level. And this is fundamentally what we need consciousness to be, to understand the extreme important of the concept of “sense of coherence” for the art and science of holistic healing.

### CONCLUSION

When small 30 μ cortical columns are taken from a random spot of cortex, no significant difference in the amount of cells can be identified. When we look at corticocortical myelinized afferents, no organization of 300 μ can be identified. When many afferents are coloured at the same time, we can see no superior columns. This means that the well known 300 μ termination columns from corticocortical afferents overlaps. Such structure does not support the existence of the discrete module proposed by Szentagothai. “The “barrels” of the rodents somatosensory cortex does not seam to be modules, and discrete modules in visual- or somatosensory cortex, does not seam to be discrete. At the cell level, the large basket cell seams to be independent of, and opposite to, a 300 μ module. The thalamus cortical afferents are shown to terminate in the fourth layer, independent of the corticocortical afferents termination column. Besides this, the most corticocortical afferents are shown to terminate laminar (so the fibres from respectively layer 7 and layer 5 of neo-cortex projects to the same layer). These considerations do not seam directly to permit any modules. Therefore, we conclude that evidence for the existence of the postulated module or column does not exist in the literature. There is no evidence for an app. 300 μ large vertically placed structure that could be able to make up for the cortical ground-unit proposed by Szentagothai.

### ACKNOWLEDGEMENTS

These studies were supported by grants from IMK Almene Fond. Our research in quality of life has been approved by the Copenhagen Scientific Ethical Committee under number (KF)V.100.2123/91.
